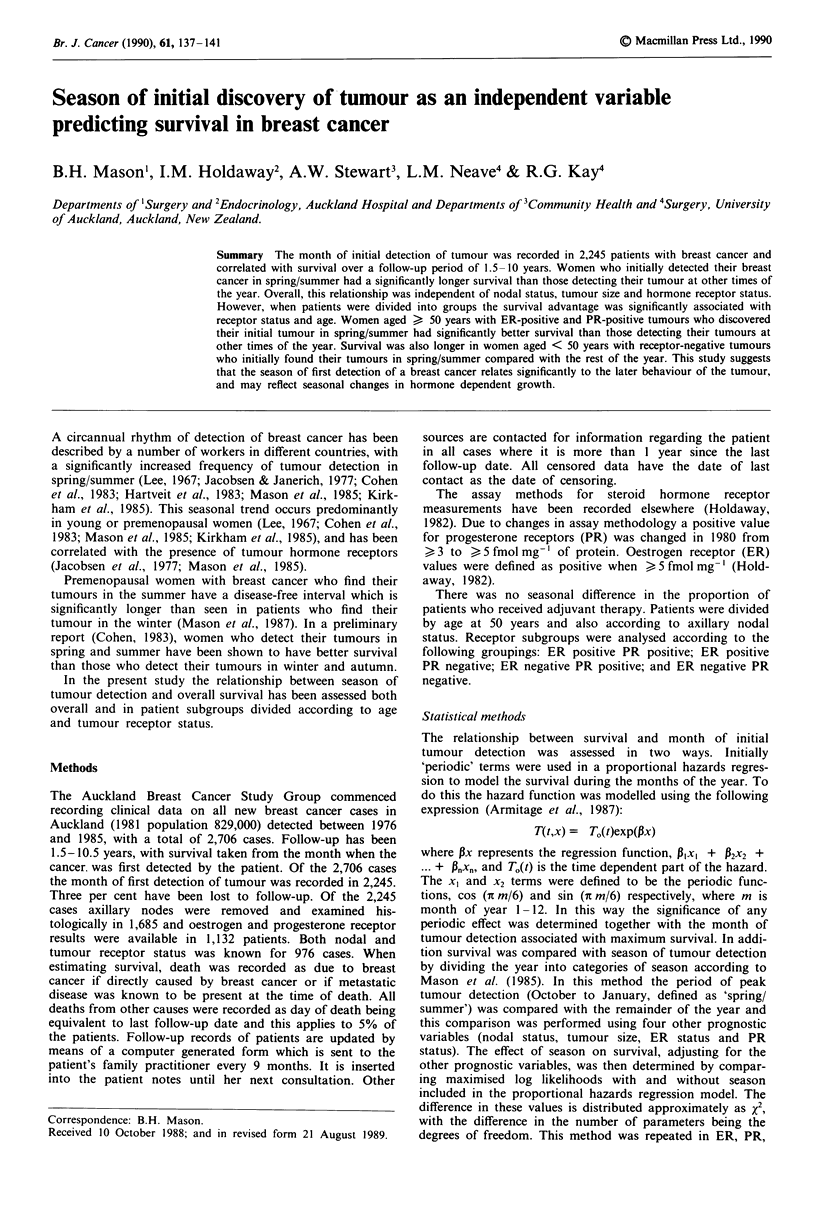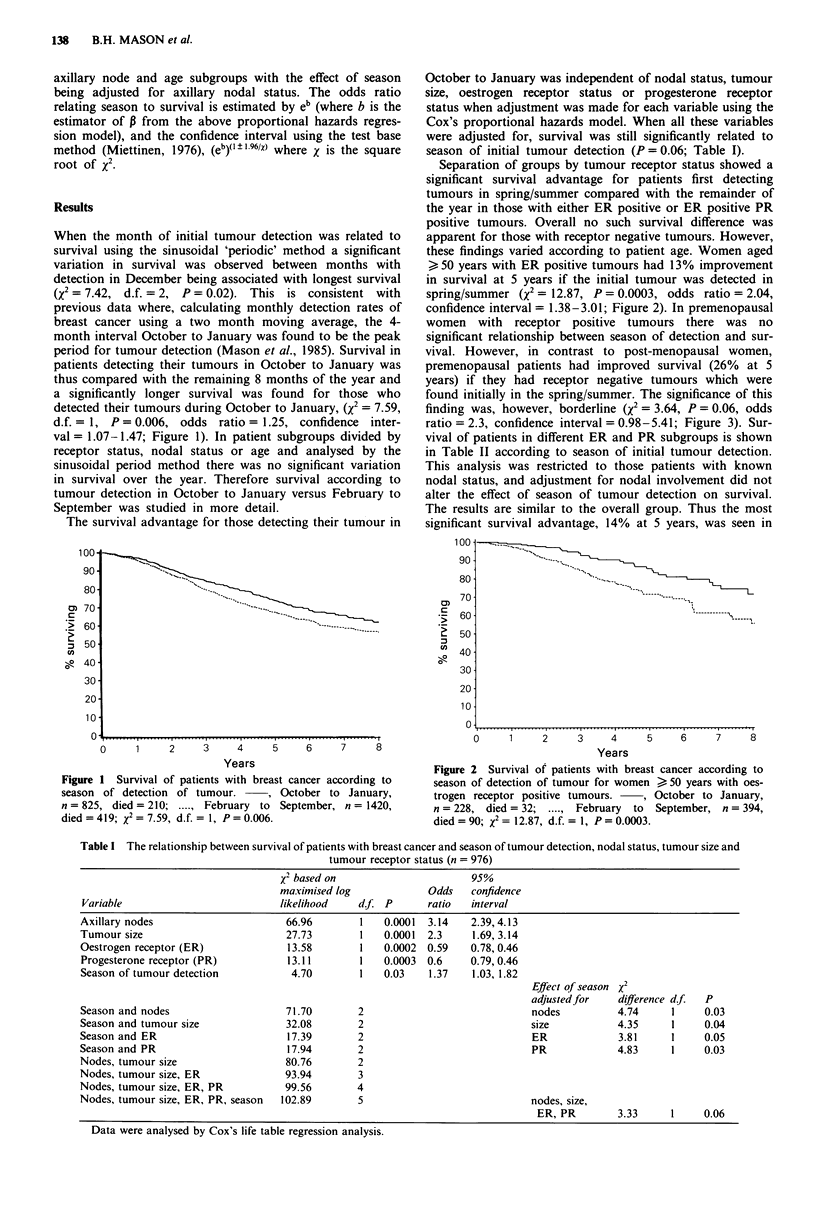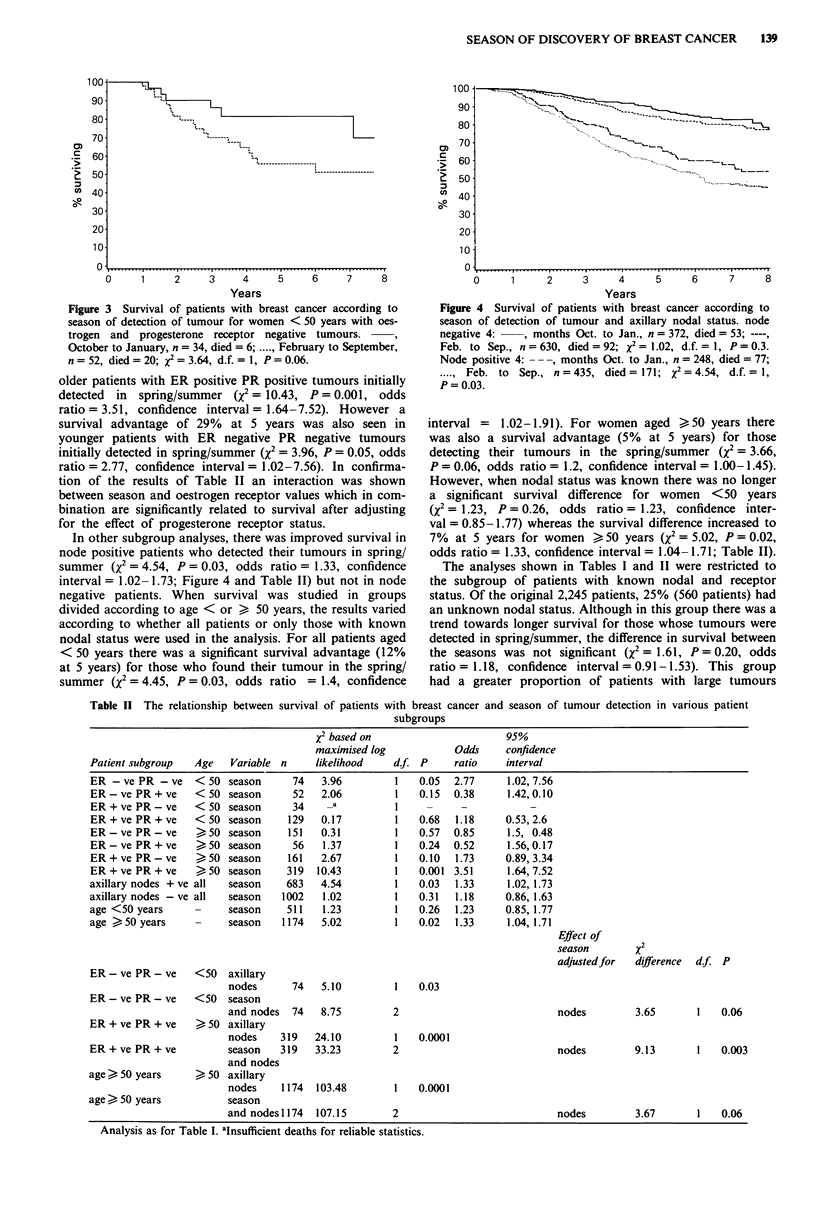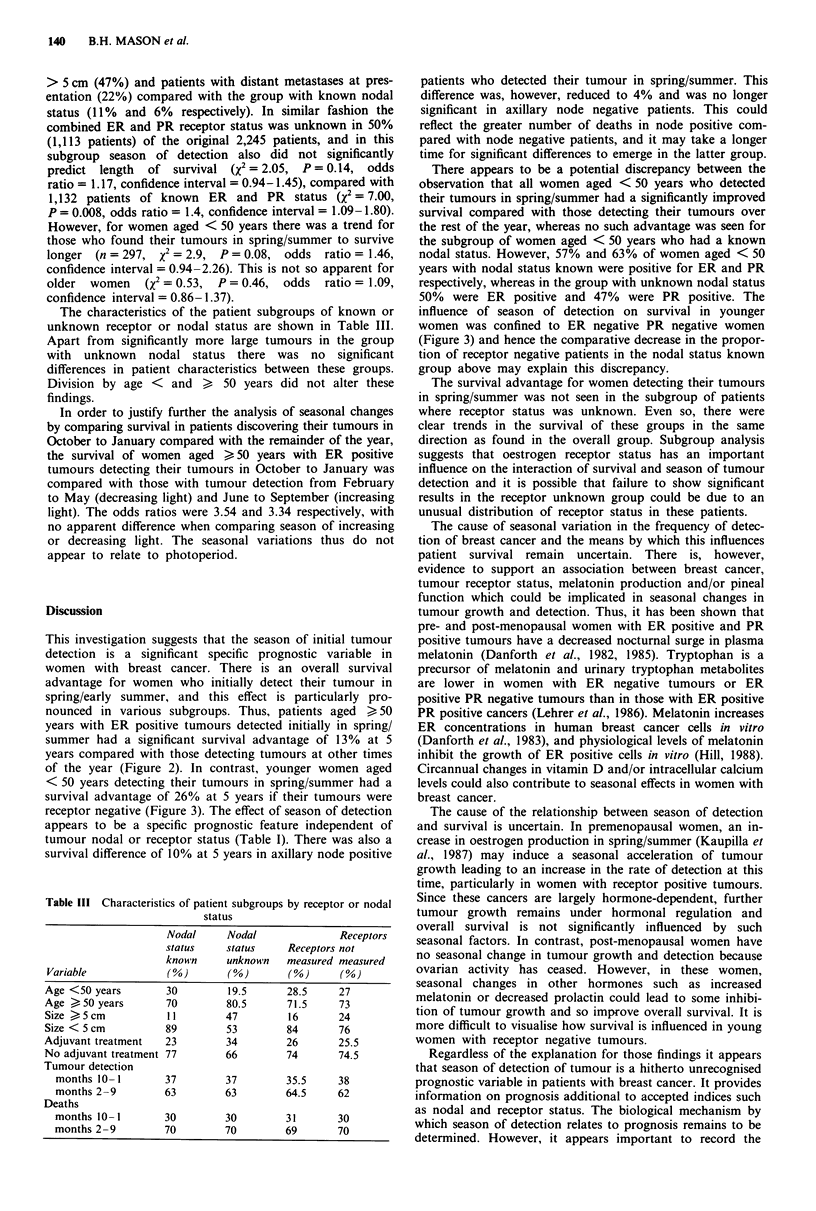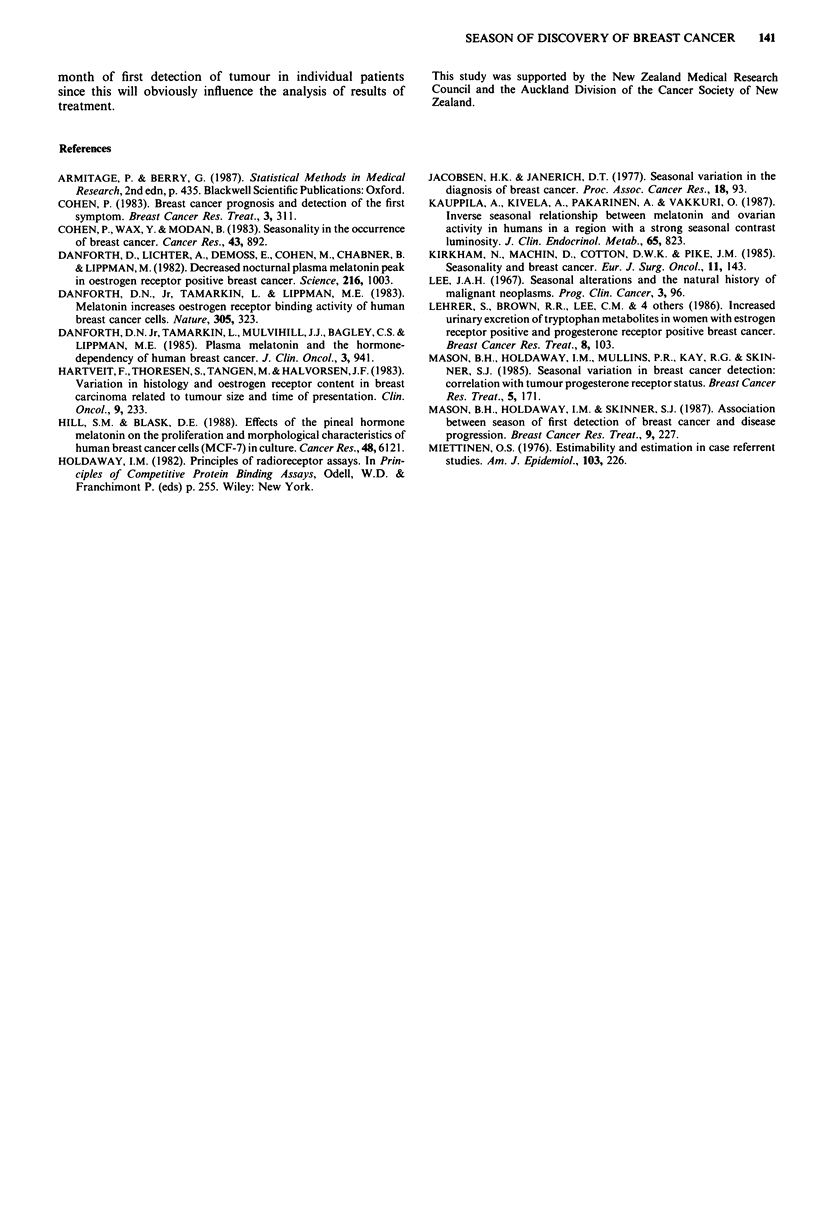# Season of initial discovery of tumour as an independent variable predicting survival in breast cancer.

**DOI:** 10.1038/bjc.1990.28

**Published:** 1990-01

**Authors:** B. H. Mason, I. M. Holdaway, A. W. Stewart, L. M. Neave, R. G. Kay

**Affiliations:** Department of Surgery, Auckland Hospital, New Zealand.

## Abstract

The month of initial detection of tumour was recorded in 2,245 patients with breast cancer and correlated with survival over a follow-up period of 1.5-10 years. Women who initially detected their breast cancer in spring/summer had a significantly longer survival than those detecting their tumour at other times of the year. Overall, this relationship was independent of nodal status, tumour size and hormone receptor status. However, when patients were divided into groups the survival advantage was significantly associated with receptor status and age. Women aged greater than or equal to 50 years with ER-positive and PR-positive tumours who discovered their initial tumour in spring/summer had significantly better survival than those detecting their tumours at other times of the year. Survival was also longer in women aged less than 50 years with receptor-negative tumours who initially found their tumours in spring/summer compared with the rest of the year. This study suggests that the season of first detection of a breast cancer relates significantly to the later behaviour of the tumour, and may reflect seasonal changes in hormone dependent growth.


*Br. J. Cancer* (1990), **61**, 137–141 © Macmillan Press Ltd., 1990

### Season of initial discovery of tumour as an independent variable predicting survival in breast cancer

B.H. Mason[1], I.M. Holdaway[2], A.W. Stewart[3], L.M. Neave[4] & R.G. Kay[4]

*Departments of [1]Surgery and [2]Endocrinology, Auckland Hospital and Departments of [3]Community Health and [4]Surgery, University of Auckland, Auckland, New Zealand.*

**Summary** The month of initial detection of tumour was recorded in 2,245 patients with breast cancer and correlated with survival over a follow-up period of 1.5–10 years. Women who initially detected their breast cancer in spring/summer had a significantly longer survival than those detecting their tumour at other times of the year. Overall, this relationship was independent of nodal status, tumour size and hormone receptor status. However, when patients were divided into groups the survival advantage was significantly associated with receptor status and age. Women aged $\geqslant$ 50 years with ER-positive and PR-positive tumours who discovered their initial tumour in spring/summer had significantly better survival than those detecting their tumours at other times of the year. Survival was also longer in women aged $<$ 50 years with receptor-negative tumours who initially found their tumours in spring/summer compared with the rest of the year. This study suggests that the season of first detection of a breast cancer relates significantly to the later behaviour of the tumour, and may reflect seasonal changes in hormone dependent growth.

A circannual rhythm of detection of breast cancer has been described by a number of workers in different countries, with a significantly increased frequency of tumour detection in spring/summer (Lee, 1967; Jacobsen & Janerich, 1977; Cohen *et al.*, 1983; Hartveit *et al.*, 1983; Mason *et al.*, 1985; Kirkham *et al.*, 1985). This seasonal trend occurs predominantly in young or premenopausal women (Lee, 1967; Cohen *et al.*, 1983; Mason *et al.*, 1985; Kirkham *et al.*, 1985), and has been correlated with the presence of tumour hormone receptors (Jacobsen *et al.*, 1977; Mason *et al.*, 1985).

Premenopausal women with breast cancer who find their tumours in the summer have a disease-free interval which is significantly longer than seen in patients who find their tumour in the winter (Mason *et al.*, 1987). In a preliminary report (Cohen, 1983), women who detect their tumours in spring and summer have been shown to have better survival than those who detect their tumours in winter and autumn.

In the present study the relationship between season of tumour detection and overall survival has been assessed both overall and in patient subgroups divided according to age and tumour receptor status.

### Methods

The Auckland Breast Cancer Study Group commenced recording clinical data on all new breast cancer cases in Auckland (1981 population 829,000) detected between 1976 and 1985, with a total of 2,706 cases. Follow-up has been 1.5–10.5 years, with survival taken from the month when the cancer was first detected by the patient. Of the 2,706 cases the month of first detection of tumour was recorded in 2,245. Three per cent have been lost to follow-up. Of the 2,245 cases axillary nodes were removed and examined histologically in 1,685 and oestrogen and progesterone receptor results were available in 1,132 patients. Both nodal and tumour receptor status was known for 976 cases. When estimating survival, death was recorded as due to breast cancer if directly caused by breast cancer or if metastatic disease was known to be present at the time of death. All deaths from other causes were recorded as day of death being equivalent to last follow-up date and this applies to 5% of the patients. Follow-up records of patients are updated by means of a computer generated form which is sent to the patient's family practitioner every 9 months. It is inserted into the patient notes until her next consultation. Other sources are contacted for information regarding the patient in all cases where it is more than 1 year since the last follow-up date. All censored data have the date of last contact as the date of censoring.

The assay methods for steroid hormone receptor measurements have been recorded elsewhere (Holdaway, 1982). Due to changes in assay methodology a positive value for progesterone receptors (PR) was changed in 1980 from $\geqslant 3$ to $\geqslant 5\ \text{fmol mg}^{-1}$ of protein. Oestrogen receptor (ER) values were defined as positive when $\geqslant 5\ \text{fmol mg}^{-1}$ (Holdaway, 1982).

There was no seasonal difference in the proportion of patients who received adjuvant therapy. Patients were divided by age at 50 years and also according to axillary nodal status. Receptor subgroups were analysed according to the following groupings: ER positive PR positive; ER positive PR negative; ER negative PR positive; and ER negative PR negative.

### *Statistical methods*

The relationship between survival and month of initial tumour detection was assessed in two ways. Initially 'periodic' terms were used in a proportional hazards regression to model the survival during the months of the year. To do this the hazard function was modelled using the following expression (Armitage *et al.*, 1987):

$$T(t,x) = T_0(t)\exp(\beta x)$$

where $\beta x$ represents the regression function, $\beta_1 x_1 + \beta_2 x_2 + \ldots + \beta_n x_n$, and $T_0(t)$ is the time dependent part of the hazard. The $x_1$ and $x_2$ terms were defined to be the periodic functions, cos $(\pi m/6)$ and sin $(\pi m/6)$ respectively, where $m$ is month of year 1–12. In this way the significance of any periodic effect was determined together with the month of tumour detection associated with maximum survival. In addition survival was compared with season of tumour detection by dividing the year into categories of season according to Mason *et al.* (1985). In this method the period of peak tumour detection (October to January, defined as 'spring/summer') was compared with the remainder of the year and this comparison was performed using four other prognostic variables (nodal status, tumour size, ER status and PR status). The effect of season on survival, adjusting for the other prognostic variables, was then determined by comparing maximised log likelihoods with and without season included in the proportional hazards regression model. The difference in these values is distributed approximately as $\chi^2$, with the difference in the number of parameters being the degrees of freedom. This method was repeated in ER, PR,

Correspondence: B.H. Mason.
Received 10 October 1988; and in revised form 21 August 1989.

axillary node and age subgroups with the effect of season being adjusted for axillary nodal status. The odds ratio relating season to survival is estimated by $e^b$ (where $b$ is the estimator of $\beta$ from the above proportional hazards regression model), and the confidence interval using the test base method (Miettinen, 1976), $(e^b)^{(1 \pm 1.96/\chi)}$ where $\chi$ is the square root of $\chi^2$.

### Results

When the month of initial tumour detection was related to survival using the sinusoidal 'periodic' method a significant variation in survival was observed between months with detection in December being associated with longest survival ($\chi^2 = 7.42$, d.f. = 2, $P = 0.02$). This is consistent with previous data where, calculating monthly detection rates of breast cancer using a two month moving average, the 4-month interval October to January was found to be the peak period for tumour detection (Mason *et al.*, 1985). Survival in patients detecting their tumours in October to January was thus compared with the remaining 8 months of the year and a significantly longer survival was found for those who detected their tumours during October to January, ($\chi^2 = 7.59$, d.f. = 1, $P = 0.006$, odds ratio = 1.25, confidence interval = 1.07–1.47; Figure 1). In patient subgroups divided by receptor status, nodal status or age and analysed by the sinusoidal period method there was no significant variation in survival over the year. Therefore survival according to tumour detection in October to January versus February to September was studied in more detail.

The survival advantage for those detecting their tumour in October to January was independent of nodal status, tumour size, oestrogen receptor status or progesterone receptor status when adjustment was made for each variable using the Cox's proportional hazards model. When all these variables were adjusted for, survival was still significantly related to season of initial tumour detection ($P = 0.06$; Table I).

Separation of groups by tumour receptor status showed a significant survival advantage for patients first detecting tumours in spring/summer compared with the remainder of the year in those with either ER positive or ER positive PR positive tumours. Overall no such survival difference was apparent for those with receptor negative tumours. However, these findings varied according to patient age. Women aged $\geqslant 50$ years with ER positive tumours had 13% improvement in survival at 5 years if the initial tumour was detected in spring/summer ($\chi^2 = 12.87$, $P = 0.0003$, odds ratio = 2.04, confidence interval = 1.38–3.01; Figure 2). In premenopausal women with receptor positive tumours there was no significant relationship between season of detection and survival. However, in contrast to post-menopausal women, premenopausal patients had improved survival (26% at 5 years) if they had receptor negative tumours which were found initially in the spring/summer. The significance of this finding was, however, borderline ($\chi^2 = 3.64$, $P = 0.06$, odds ratio = 2.3, confidence interval = 0.98–5.41; Figure 3). Survival of patients in different ER and PR subgroups is shown in Table II according to season of initial tumour detection. This analysis was restricted to those patients with known nodal status, and adjustment for nodal involvement did not alter the effect of season of tumour detection on survival. The results are similar to the overall group. Thus the most significant survival advantage, 14% at 5 years, was seen in

**Figure 1** Survival of patients with breast cancer according to season of detection of tumour. ——, October to January, $n = 825$, died = 210; ....., February to September, $n = 1420$, died = 419; $\chi^2 = 7.59$, d.f. = 1, $P = 0.006$.

**Figure 2** Survival of patients with breast cancer according to season of detection of tumour for women $\geqslant 50$ years with oestrogen receptor positive tumours. ——, October to January, $n = 228$, died = 32; ....., February to September, $n = 394$, died = 90; $\chi^2 = 12.87$, d.f. = 1, $P = 0.0003$.

**Table I** The relationship between survival of patients with breast cancer and season of tumour detection, nodal status, tumour size and tumour receptor status ($n = 976$)

| *Variable* | $\chi^2$ *based on maximised log likelihood* | *d.f.* | *P* | *Odds ratio* | *95% confidence interval* | *Effect of season adjusted for* | $\chi^2$ *difference* | *d.f.* | *P* |
|---|---|---|---|---|---|---|---|---|---|
| Axillary nodes | 66.96 | 1 | 0.0001 | 3.14 | 2.39, 4.13 | | | | |
| Tumour size | 27.73 | 1 | 0.0001 | 2.3 | 1.69, 3.14 | | | | |
| Oestrogen receptor (ER) | 13.58 | 1 | 0.0002 | 0.59 | 0.78, 0.46 | | | | |
| Progesterone receptor (PR) | 13.11 | 1 | 0.0003 | 0.6 | 0.79, 0.46 | | | | |
| Season of tumour detection | 4.70 | 1 | 0.03 | 1.37 | 1.03, 1.82 | | | | |
| Season and nodes | 71.70 | 2 | | | | nodes | 4.74 | 1 | 0.03 |
| Season and tumour size | 32.08 | 2 | | | | size | 4.35 | 1 | 0.04 |
| Season and ER | 17.39 | 2 | | | | ER | 3.81 | 1 | 0.05 |
| Season and PR | 17.94 | 2 | | | | PR | 4.83 | 1 | 0.03 |
| Nodes, tumour size | 80.76 | 2 | | | | | | | |
| Nodes, tumour size, ER | 93.94 | 3 | | | | | | | |
| Nodes, tumour size, ER, PR | 99.56 | 4 | | | | | | | |
| Nodes, tumour size, ER, PR, season | 102.89 | 5 | | | | nodes, size, ER, PR | 3.33 | 1 | 0.06 |

Data were analysed by Cox's life table regression analysis.

**Figure 3** Survival of patients with breast cancer according to season of detection of tumour for women < 50 years with oestrogen and progesterone receptor negative tumours. ——, October to January, $n = 34$, died = 6; ....., February to September, $n = 52$, died = 20; $\chi^2 = 3.64$, d.f. = 1, $P = 0.06$.

**Figure 4** Survival of patients with breast cancer according to season of detection of tumour and axillary nodal status. node negative 4: ——, months Oct. to Jan., $n = 372$, died = 53; ----, Feb. to Sep., $n = 630$, died = 92; $\chi^2 = 1.02$, d.f. = 1, $P = 0.3$. Node positive 4: – – –, months Oct. to Jan., $n = 248$, died = 77; ....., Feb. to Sep., $n = 435$, died = 171; $\chi^2 = 4.54$, d.f. = 1, $P = 0.03$.

older patients with ER positive PR positive tumours initially detected in spring/summer ($\chi^2 = 10.43$, $P = 0.001$, odds ratio = 3.51, confidence interval = 1.64–7.52). However a survival advantage of 29% at 5 years was also seen in younger patients with ER negative PR negative tumours initially detected in spring/summer ($\chi^2 = 3.96$, $P = 0.05$, odds ratio = 2.77, confidence interval = 1.02–7.56). In confirmation of the results of Table II an interaction was shown between season and oestrogen receptor values which in combination are significantly related to survival after adjusting for the effect of progesterone receptor status.

In other subgroup analyses, there was improved survival in node positive patients who detected their tumours in spring/summer ($\chi^2 = 4.54$, $P = 0.03$, odds ratio = 1.33, confidence interval = 1.02–1.73; Figure 4 and Table II) but not in node negative patients. When survival was studied in groups divided according to age < or ⩾ 50 years, the results varied according to whether all patients or only those with known nodal status were used in the analysis. For all patients aged < 50 years there was a significant survival advantage (12% at 5 years) for those who found their tumour in the spring/summer ($\chi^2 = 4.45$, $P = 0.03$, odds ratio = 1.4, confidence interval = 1.02–1.91). For women aged ⩾50 years there was also a survival advantage (5% at 5 years) for those detecting their tumours in the spring/summer ($\chi^2 = 3.66$, $P = 0.06$, odds ratio = 1.2, confidence interval = 1.00–1.45). However, when nodal status was known there was no longer a significant survival difference for women <50 years ($\chi^2 = 1.23$, $P = 0.26$, odds ratio = 1.23, confidence interval = 0.85–1.77) whereas the survival difference increased to 7% at 5 years for women ⩾50 years ($\chi^2 = 5.02$, $P = 0.02$, odds ratio = 1.33, confidence interval = 1.04–1.71; Table II).

The analyses shown in Tables I and II were restricted to the subgroup of patients with known nodal and receptor status. Of the original 2,245 patients, 25% (560 patients) had an unknown nodal status. Although in this group there was a trend towards longer survival for those whose tumours were detected in spring/summer, the difference in survival between the seasons was not significant ($\chi^2 = 1.61$, $P = 0.20$, odds ratio = 1.18, confidence interval = 0.91–1.53). This group had a greater proportion of patients with large tumours

**Table II** The relationship between survival of patients with breast cancer and season of tumour detection in various patient subgroups

| *Patient subgroup* | *Age* | *Variable* | *n* | *$\chi^2$ based on maximised log likelihood* | *d.f.* | *P* | *Odds ratio* | *95% confidence interval* | | | | |
|---|---|---|---|---|---|---|---|---|---|---|---|---|
| ER – ve PR – ve | < 50 | season | 74 | 3.96 | 1 | 0.05 | 2.77 | 1.02, 7.56 | | | | |
| ER – ve PR + ve | < 50 | season | 52 | 2.06 | 1 | 0.15 | 0.38 | 1.42, 0.10 | | | | |
| ER + ve PR – ve | < 50 | season | 34 | –[a] | 1 | – | – | – | | | | |
| ER + ve PR + ve | < 50 | season | 129 | 0.17 | 1 | 0.68 | 1.18 | 0.53, 2.6 | | | | |
| ER – ve PR – ve | ⩾ 50 | season | 151 | 0.31 | 1 | 0.57 | 0.85 | 1.5, 0.48 | | | | |
| ER – ve PR + ve | ⩾ 50 | season | 56 | 1.37 | 1 | 0.24 | 0.52 | 1.56, 0.17 | | | | |
| ER + ve PR – ve | ⩾ 50 | season | 161 | 2.67 | 1 | 0.10 | 1.73 | 0.89, 3.34 | | | | |
| ER + ve PR + ve | ⩾ 50 | season | 319 | 10.43 | 1 | 0.001 | 3.51 | 1.64, 7.52 | | | | |
| axillary nodes + ve | all | season | 683 | 4.54 | 1 | 0.03 | 1.33 | 1.02, 1.73 | | | | |
| axillary nodes – ve | all | season | 1002 | 1.02 | 1 | 0.31 | 1.18 | 0.86, 1.63 | | | | |
| age <50 years | – | season | 511 | 1.23 | 1 | 0.26 | 1.23 | 0.85, 1.77 | | | | |
| age ⩾ 50 years | – | season | 1174 | 5.02 | 1 | 0.02 | 1.33 | 1.04, 1.71 | | | | |
| | | | | | | | | | *Effect of season adjusted for* | *$\chi^2$ difference* | *d.f.* | *P* |
| ER – ve PR – ve | <50 | axillary nodes | 74 | 5.10 | 1 | 0.03 | | | | | | |
| ER – ve PR – ve | <50 | season and nodes | 74 | 8.75 | 2 | | | | nodes | 3.65 | 1 | 0.06 |
| ER + ve PR + ve | ⩾ 50 | axillary nodes | 319 | 24.10 | 1 | 0.0001 | | | | | | |
| ER + ve PR + ve | | season and nodes | 319 | 33.23 | 2 | | | | nodes | 9.13 | 1 | 0.003 |
| age ⩾ 50 years | ⩾ 50 | axillary nodes | 1174 | 103.48 | 1 | 0.0001 | | | | | | |
| age ⩾ 50 years | | season and nodes | 1174 | 107.15 | 2 | | | | nodes | 3.67 | 1 | 0.06 |

Analysis as for Table I. [a]Insufficient deaths for reliable statistics.

> 5 cm (47%) and patients with distant metastases at presentation (22%) compared with the group with known nodal status (11% and 6% respectively). In similar fashion the combined ER and PR receptor status was unknown in 50% (1,113 patients) of the original 2,245 patients, and in this subgroup season of detection also did not significantly predict length of survival ($\chi^2 = 2.05$, $P = 0.14$, odds ratio = 1.17, confidence interval = 0.94–1.45), compared with 1,132 patients of known ER and PR status ($\chi^2 = 7.00$, $P = 0.008$, odds ratio = 1.4, confidence interval = 1.09–1.80). However, for women aged < 50 years there was a trend for those who found their tumours in spring/summer to survive longer ($n = 297$, $\chi^2 = 2.9$, $P = 0.08$, odds ratio = 1.46, confidence interval = 0.94–2.26). This is not so apparent for older women ($\chi^2 = 0.53$, $P = 0.46$, odds ratio = 1.09, confidence interval = 0.86–1.37).

The characteristics of the patient subgroups of known or unknown receptor or nodal status are shown in Table III. Apart from significantly more large tumours in the group with unknown nodal status there was no significant differences in patient characteristics between these groups. Division by age < and ⩾ 50 years did not alter these findings.

In order to justify further the analysis of seasonal changes by comparing survival in patients discovering their tumours in October to January compared with the remainder of the year, the survival of women aged ⩾50 years with ER positive tumours detecting their tumours in October to January was compared with those with tumour detection from February to May (decreasing light) and June to September (increasing light). The odds ratios were 3.54 and 3.34 respectively, with no apparent difference when comparing season of increasing or decreasing light. The seasonal variations thus do not appear to relate to photoperiod.

### Discussion

This investigation suggests that the season of initial tumour detection is a significant specific prognostic variable in women with breast cancer. There is an overall survival advantage for women who initially detect their tumour in spring/early summer, and this effect is particularly pronounced in various subgroups. Thus, patients aged ⩾50 years with ER positive tumours detected initially in spring/summer had a significant survival advantage of 13% at 5 years compared with those detecting tumours at other times of the year (Figure 2). In contrast, younger women aged < 50 years detecting their tumours in spring/summer had a survival advantage of 26% at 5 years if their tumours were receptor negative (Figure 3). The effect of season of detection appears to be a specific prognostic feature independent of tumour nodal or receptor status (Table I). There was also a survival difference of 10% at 5 years in axillary node positive patients who detected their tumour in spring/summer. This difference was, however, reduced to 4% and was no longer significant in axillary node negative patients. This could reflect the greater number of deaths in node positive compared with node negative patients, and it may take a longer time for significant differences to emerge in the latter group.

There appears to be a potential discrepancy between the observation that all women aged < 50 years who detected their tumours in spring/summer had a significantly improved survival compared with those detecting their tumours over the rest of the year, whereas no such advantage was seen for the subgroup of women aged < 50 years who had a known nodal status. However, 57% and 63% of women aged < 50 years with nodal status known were positive for ER and PR respectively, whereas in the group with unknown nodal status 50% were ER positive and 47% were PR positive. The influence of season of detection on survival in younger women was confined to ER negative PR negative women (Figure 3) and hence the comparative decrease in the proportion of receptor negative patients in the nodal status known group above may explain this discrepancy.

The survival advantage for women detecting their tumours in spring/summer was not seen in the subgroup of patients where receptor status was unknown. Even so, there were clear trends in the survival of these groups in the same direction as found in the overall group. Subgroup analysis suggests that oestrogen receptor status has an important influence on the interaction of survival and season of tumour detection and it is possible that failure to show significant results in the receptor unknown group could be due to an unusual distribution of receptor status in these patients.

The cause of seasonal variation in the frequency of detection of breast cancer and the means by which this influences patient survival remain uncertain. There is, however, evidence to support an association between breast cancer, tumour receptor status, melatonin production and/or pineal function which could be implicated in seasonal changes in tumour growth and detection. Thus, it has been shown that pre- and post-menopausal women with ER positive and PR positive tumours have a decreased nocturnal surge in plasma melatonin (Danforth *et al.*, 1982, 1985). Tryptophan is a precursor of melatonin and urinary tryptophan metabolites are lower in women with ER negative tumours or ER positive PR negative tumours than in those with ER positive PR positive cancers (Lehrer *et al.*, 1986). Melatonin increases ER concentrations in human breast cancer cells *in vitro* (Danforth *et al.*, 1983), and physiological levels of melatonin inhibit the growth of ER positive cells *in vitro* (Hill, 1988). Circannual changes in vitamin D and/or intracellular calcium levels could also contribute to seasonal effects in women with breast cancer.

The cause of the relationship between season of detection and survival is uncertain. In premenopausal women, an increase in oestrogen production in spring/summer (Kaupilla *et al.*, 1987) may induce a seasonal acceleration of tumour growth leading to an increase in the rate of detection at this time, particularly in women with receptor positive tumours. Since these cancers are largely hormone-dependent, further tumour growth remains under hormonal regulation and overall survival is not significantly influenced by such seasonal factors. In contrast, post-menopausal women have no seasonal change in tumour growth and detection because ovarian activity has ceased. However, in these women, seasonal changes in other hormones such as increased melatonin or decreased prolactin could lead to some inhibition of tumour growth and so improve overall survival. It is more difficult to visualise how survival is influenced in young women with receptor negative tumours.

Regardless of the explanation for those findings it appears that season of detection of tumour is a hitherto unrecognised prognostic variable in patients with breast cancer. It provides information on prognosis additional to accepted indices such as nodal and receptor status. The biological mechanism by which season of detection relates to prognosis remains to be determined. However, it appears important to record the

**Table III** Characteristics of patient subgroups by receptor or nodal status

| *Variable* | *Nodal status known (%)* | *Nodal status unknown (%)* | *Receptors measured (%)* | *Receptors not measured (%)* |
|---|---|---|---|---|
| Age <50 years | 30 | 19.5 | 28.5 | 27 |
| Age ⩾50 years | 70 | 80.5 | 71.5 | 73 |
| Size ⩾5 cm | 11 | 47 | 16 | 24 |
| Size < 5 cm | 89 | 53 | 84 | 76 |
| Adjuvant treatment | 23 | 34 | 26 | 25.5 |
| No adjuvant treatment | 77 | 66 | 74 | 74.5 |
| Tumour detection | | | | |
| months 10–1 | 37 | 37 | 35.5 | 38 |
| months 2–9 | 63 | 63 | 64.5 | 62 |
| Deaths | | | | |
| months 10–1 | 30 | 30 | 31 | 30 |
| months 2–9 | 70 | 70 | 69 | 70 |

month of first detection of tumour in individual patients since this will obviously influence the analysis of results of treatment.

This study was supported by the New Zealand Medical Research Council and the Auckland Division of the Cancer Society of New Zealand.

**References**

ARMITAGE, P. & BERRY, G. (1987). *Statistical Methods in Medical Research*, 2nd edn, p. 435. Blackwell Scientific Publications: Oxford.

COHEN, P. (1983). Breast cancer prognosis and detection of the first symptom. *Breast Cancer Res. Treat.*, **3,** 311.

COHEN, P., WAX, Y. & MODAN, B. (1983). Seasonality in the occurrence of breast cancer. *Cancer Res.*, **43,** 892.

DANFORTH, D., LICHTER, A., DEMOSS, E., COHEN, M., CHABNER, B. & LIPPMAN, M. (1982). Decreased nocturnal plasma melatonin peak in oestrogen receptor positive breast cancer. *Science*, **216,** 1003.

DANFORTH, D.N., Jr, TAMARKIN, L. & LIPPMAN, M.E. (1983). Melatonin increases oestrogen receptor binding activity of human breast cancer cells. *Nature*, **305,** 323.

DANFORTH, D.N. Jr, TAMARKIN, L., MULVIHILL, J.J., BAGLEY, C.S. & LIPPMAN, M.E. (1985). Plasma melatonin and the hormone-dependency of human breast cancer. *J. Clin. Oncol.*, **3,** 941.

HARTVEIT, F., THORESEN, S., TANGEN, M. & HALVORSEN, J.F. (1983). Variation in histology and oestrogen receptor content in breast carcinoma related to tumour size and time of presentation. *Clin. Oncol.*, **9,** 233.

HILL, S.M. & BLASK, D.E. (1988). Effects of the pineal hormone melatonin on the proliferation and morphological characteristics of human breast cancer cells (MCF-7) in culture. *Cancer Res.*, **48,** 6121.

HOLDAWAY, I.M. (1982). Principles of radioreceptor assays. In *Principles of Competitive Protein Binding Assays*, Odell, W.D. & Franchimont P. (eds) p. 255. Wiley: New York.

JACOBSEN, H.K. & JANERICH, D.T. (1977). Seasonal variation in the diagnosis of breast cancer. *Proc. Assoc. Cancer Res.*, **18,** 93.

KAUPPILA, A., KIVELA, A., PAKARINEN, A. & VAKKURI, O. (1987). Inverse seasonal relationship between melatonin and ovarian activity in humans in a region with a strong seasonal contrast luminosity. *J. Clin. Endocrinol. Metab.*, **65,** 823.

KIRKHAM, N., MACHIN, D., COTTON, D.W.K. & PIKE, J.M. (1985). Seasonality and breast cancer. *Eur. J. Surg. Oncol.*, **11,** 143.

LEE, J.A.H. (1967). Seasonal alterations and the natural history of malignant neoplasms. *Prog. Clin. Cancer*, **3,** 96.

LEHRER, S., BROWN, R.R., LEE, C.M. & 4 others (1986). Increased urinary excretion of tryptophan metabolites in women with estrogen receptor positive and progesterone receptor positive breast cancer. *Breast Cancer Res. Treat.*, **8,** 103.

MASON, B.H., HOLDAWAY, I.M., MULLINS, P.R., KAY, R.G. & SKINNER, S.J. (1985). Seasonal variation in breast cancer detection: correlation with tumour progesterone receptor status. *Breast Cancer Res. Treat.*, **5,** 171.

MASON, B.H., HOLDAWAY, I.M. & SKINNER, S.J. (1987). Association between season of first detection of breast cancer and disease progression. *Breast Cancer Res. Treat.*, **9,** 227.

MIETTINEN, O.S. (1976). Estimability and estimation in case referrent studies. *Am. J. Epidemiol.*, **103,** 226.